# Supplementary material for: Integrating anamnestic and lifestyle data with sphingolipid levels for risk-based prostate cancer screening
Source: J Transl Med. 2025 Jul 14;23:790. doi: 10.1186/s12967-025-06820-9 (PMC12257792; doi:10.1186/s12967-025-06820-9)
Supplement: Supplementary file 2 — Supplementary Material 2 [file 12967_2025_6820_MOESM2_ESM.docx]

Giovanna Chiorino

Cancer Genomics Laboratory

Fondazione Edo ed Elvo Tempia

Via Malta 3, 13900 Biella – ITALY

Email: giovanna.chiorino@fondazionetempia.org

Phone number: +39 015351830

Fax: +39 01521116

June 25^ft^, 2025

To Dr Michael Liebman

Editor

*Journal of Translational Medicine*

Dear Editor,

We are pleased to submit our revised manuscript entitled "**Integrating anamnestic and lifestyle data with sphingolipid levels for risk-based prostate cancer screening**" for consideration for publication in the Journal of Translational Medicine.

We have replied to all the comments of the referees.

Thank you for your time and consideration. We look forward to hearing from you at your earliest convenience.

Sincerely,

Giovanna Chiorino, PhD

Genomics Lab, Fondazione Edo ed Elvo Tempia, 13900 Biella, Italy

[giovanna.chiorino@fondazionetempia.org](mailto:giovanna.chiorino@fondazionetempia.org)
